# Evaluation of the international Ki67 working group cut point recommendations for early breast cancer: comparison with 21-gene assay results in a large integrated health care system

**DOI:** 10.1007/s10549-023-07118-4

**Published:** 2023-10-17

**Authors:** Veronica C. Shim, Robin J. Baker, Wen Jing, Roisin Puentes, Sally S. Agersborg, Thomas K. Lee, Wamda GoreaI, Ninah Achacoso, Catherine Lee, Marvella Villasenor, Amy Lin, Malathy Kapali, Laurel A. Habel

**Affiliations:** 1 The Permanente Medical Group, Northern California Kaiser Permanente, Oakland, CA USA; 2 The Permanente Medical Group, Northern California Kaiser Permanente, San Francisco, CA USA; 3 The Permanente Medicine, Northern California Kaiser Permanente, San Francisco, CA USA; 4 NeoGenomics Laboratories, Inc., Aliso Viejo, CA USA; 5 The Division of Research, Northern California Kaiser Permanente, 2000 Broadway, Oakland, CA 94612 USA; 6 The Permanente Medical Group, Northern California Kaiser Permanente, Sacramento, CA USA

**Keywords:** Breast cancer, Ki67, Immunohistochemistry, Reproducibility, Prognosis

## Abstract

**Purpose:**

The International Ki67 Working Group (IKWG) has developed training for immunohistochemistry (IHC) scoring reproducibility and recommends cut points of ≤ 5% and ≥ 30% for prognosis in ER+, HER2−, stage I/II breast cancer. We examined scoring reproducibility following IKWG training and evaluated these cut points for selecting patients for further testing with the 21-gene Recurrence Score (RS) assay.

**Methods:**

We included 307 women aged 50+ years with node-negative, ER+PR+HER2− breast cancer and with available RS results. Slides from the diagnostic biopsy were stained for Ki67 and scored using digital image analysis (IA). Two IHC pathologists underwent IKWG training and visually scored slides, blinded to each other and IA readings. Interobserver reproducibility was examined using intraclass correlation (ICC) and Kappa statistics.

**Results:**

Depending on reader, 8.8–16.0% of our cohort had Ki67 ≤ 5% and 11.4–22.5% had scores ≥ 30%. The ICC for Ki67 scores by the two pathologists was 0.82 (95% CI 0.78–0.85); it was 0.79 (95% CI 0.74–0.83) for pathologist 1 and IA and 0.76 (95% CI 0.71–0.80) for pathologist 2 and IA. For Ki67 scores ≤ 5%, the percentages with RS < 26 were 92.6%, 91.8%, and 90.9% for pathologist 1, pathologist 2, and IA, respectively. For Ki67 scores ≥ 30%, the percentages with RS ≥ 26 were 41.5%, 51.4%, and 27.5%, respectively.

**Conclusion:**

The IKWG’s Ki67 training resulted in moderate to strong reproducibility across readers but cut points had only moderate overlap with RS cut points, especially for Ki67 ≥ 30% and RS ≥ 26; thus, their clinical utility for a 21-gene assay testing pathway remains unclear.

## Introduction

While considered an established marker of cell proliferation for decades, the potential role for Ki67 immunohistochemistry (IHC) in breast cancer management has remained unclear due largely to its high inter-observer variability and the lack of established cutoff points for clinical decisions [1]. Nonetheless, Ki67 has been used in several clinical trials (e.g., POETIC) [2]. In 2021, it was FDA approved as a companion diagnostic for selection of high-risk patients for treatment with CDK4/6 inhibitors, although inter-observer reproducibility remained a concern [3] and in 2023, FDA removed the Ki-67 testing requirement [4].

In 2021, the Ki67 International Working Group (IKWG) published updated recommendations for standardizing the visual assessment of Ki67 IHC in breast tissue [5]. In addition to scoring methods, recommendations included that breast cancer samples for Ki67 testing be processed in line with American Society of Clinical Oncology and the College of American Pathologists (ASCO/CAP) guidelines for HER2 and hormone receptors, and that they ideally be tested on core needle biopsies since this minimizes fixation problems that can impact analytical validity. When following their analytic and scoring guidelines, the IKWG concluded that Ki67 IHC cut points of ≤ 5% and ≥ 30% have sufficient clinical utility for patients with ER+HER2− stage I/II breast cancer and can be used to identify patients who can avoid or proceed with chemotherapy.

Multiple studies [6–11] have examined the extent to which Ki67 IHC correlates with the 21-gene breast cancer recurrence score (RS) assay, which is included in NCCN and ASCO guidelines for selecting HR+HER2− breast cancer patients for chemotherapy [12, 13]. There also has been significant interest in whether Ki67 IHC alone or together with other IHC markers or clinical factors could be used as a cost-effective surrogate for the 21-gene assay or to identify a subset of patients who could avoid this or other multigene tests [14, 15]. However, these studies have used various scoring methods and cut points for Ki67 IHC, have typically used surgical specimens for both Ki67 and the 21-gene assay, and have often not been restricted to patients intended for the recent IKWG recommendation (i.e., patients with ER+HER2− stage I/II breast cancer).

The goal of our study was to follow the recent IKWG’s visual assessment guidelines and examine Ki67 IHC scoring reproducibility in a real-world setting. We also evaluated whether Ki67 IHC cut-off points (≤ 5%, ≥ 30%) could accurately identify patients with either low 21-gene RS (< 26) or high RS (≥ 26) among a clinically low-risk group of early-stage breast cancer patients, who we defined as women aged 50 + years diagnosed with ER+PR+HER2−, node-negative disease. In addition, we examined Ki67 scoring results done by image analysis (IA) and Ki67 cut points used in other studies and patient populations.

## Study design and methods

### Setting and source population

The study was conducted within Kaiser Permanente Northern California (KPNC), an integrated health care system providing comprehensive primary and specialty care to approximately 4.4 million members at 21 hospitals. Approximately 3500 enrollees are diagnosed with a new invasive breast cancer each year. Following ASCO/CAP guidelines, all diagnostic biopsies are processed, and slides undergo IHC staining for ER, PR, and HER2 at local pathology departments and are sent to a central IHC laboratory for scoring. Ki67 is not routinely done in breast cancer because Ki67 has not been clinically relevant in the treatment decisions in KPNC. The KPNC pathology departments and laboratories are certified under the Clinical Laboratory Improvement Amendments (CLIA) of 1988. During the study period, all breast IHC scoring was done by one of three pathologists specializing in semiquantitative IHC. Patients with ER+HER2− disease may have surgical specimens sent to Exact Sciences for testing by the 21-gene recurrence score assay to guide chemotherapy treatment decisions, as recommended by NCCN [13]. From January 2018 through December 2020, approximately 3000 patients aged 50+ years had testing by the 21-gene recurrence score assay. Other multi-gene tests for chemotherapy decisions (e.g., Mammoprint) are rarely ordered within KPNC. The study was approved by the KPNC IRB; patient consent was waived.

### Study patients

We included a simple random sample of all women aged 50+ years diagnosed from 2018 to 2020 with lymph node-negative, ER+PR+HER2− invasive breast cancer and whose tumors (from surgery) had undergone testing by the 21- gene recurrence score assay. The sample size was based on both resource constraints and on the hypothesized number needed to address aims (*n* ~ 300). Of the 320 patients we initially randomly selected, tissue was unavailable on 13, leaving a final study population of 307 patients.

### Ki67 staining, training, and scoring

We followed the recommendations of the IKWG for studies of Ki67 with respect to type (core biopsy) and age (< 5 years) of tissue specimen and visual scoring methods [5]. Archived blocks with core biopsy specimens that had been used for the original IHC testing for ER, PR, and HER2 were identified and retrieved. The blocks were sent to NeoGenomics Laboratories, Inc for IHC staining for Ki67 (Dako clone MIB1), slides scanned at 20× using Leica Systems Aperio AT2 scanner, and scoring performed by image analysis (IA) algorithm validated at NeoGenomics Laboratories for clinical use, using the hot spot counting method of 500–1000 cells [16]. Image analysis performed using 40× digital probe set in at least 3 areas of hot spots for Ki-67 values as percentage positive cells and intensity by two pathologists (authors TKL and WG). This laboratory is CLIA certified to perform high complexity clinical laboratory testing. The study also included two KPNC immunopathologists (authors RJB and WJ) who each scored 1400–3000 breast cases per year from 2018 to 2020. Both immunopathologists underwent the web-based IKWG calibration training (see http://www.gpec.ubc.ca/calibrator) and independently visually scored all slides using the global counting method (400 cells), blinded to each other and to readings by IA. Weighted and unweighted Ki67 scores were generated. This calibrator is publicly accessible at http://www.gpec.ubc.ca/calibrator. The detailed scoring protocol was found in Supplementary Document: Instructions for Ki-67 Reproducibility Study Phase 3: Core Biopsies.

### Analytic methods

We compared Ki67 scores and examined inter-rater reliability across pathologists using intraclass correlation (ICC) and Kappa statistics. We also determined the percent of patients with low Ki67 scores (≤ 5) by each pathologist and by IA who also had low RS (< 26) and the percent with Ki67 scores ≥ 30 who also had high RS (≥ 26). In secondary analyses, we examined other Ki67 cut points and subgroups of patients based on a combination of PR scores and/or tumor grade.

## Results

Selected characteristics of the study population are provided in Table 1. In this low-risk population of breast cancer patients, there were 40 patients (or 13%) with a high (≥ 26) RS. The percent of women with high RS was similar across racial/ethnic groups, but a higher percent of younger women, and women with larger and higher-grade tumors had RS ≥ 26. In addition, a substantially higher percent of women with low ER or PR (i.e., 1–9% staining) had RS ≥ 26.

**Table 1:** Selected characteristics of breast cancer cases, by 21 gene recurrence score

Characteristic	Overall *N* = 307 (Column %)	RS < 26 *N* = 267 (Row %)	RS ≥ 26 *N* = 40 (Row %)
Age at dx			
50–59	113 (36.8)	94 (83.2)	19 (16.8)
60–69	142 (46.3)	126 (88.7)	16 (11.3)
70–79	50 (16.3)	45 (90.0)	5 (10.0)
80+	2 (0.7)	2 (100.0)	0
Race			
Asian	61 (19.9)	52 (85.2)	9 (14.8)
Black	10 (3.3)	9 (90.0)	1 (10.0)
Hispanic	34 (11.1)	29 (85.3)	5 (14.7)
Non-Hispanic white	189 (61.6)	165 (87.3)	24 (12.7)
Other/unknown	12 (3.9)	11 (91.7)	1 (8.3)
Tumor size			
< 2 cm (T1)	213 (69.4)	192 (90.1)	21 (9.9)
2–5.0 cm (T2)	86 (28.0)	69 (80.2)	17 (19.8)
5.1 + cm (T3)	8 (2.6)	6 (75.0)	2 (25.0)
Tumor grade (bx)			
Low	109 (35.5)	106 (97.2)	3 (2.8)
Intermediate	166 (54.1)	143 (86.1)	23 (13.9)
High	32 (10.4)	18 (56.2)	14 (43.8)
ER			
1–9	3 (1.0)	1 (33.3)	2 (66.7)
10+	304 (99.0)	266 (87.5)	38 (12.5)
PR			
1–9	51 (16.6)	30 (58.8)	21 (41.2)
10+	256 (83.4)	237 (92.6)	19 (7.4)

**Fig. 1:**
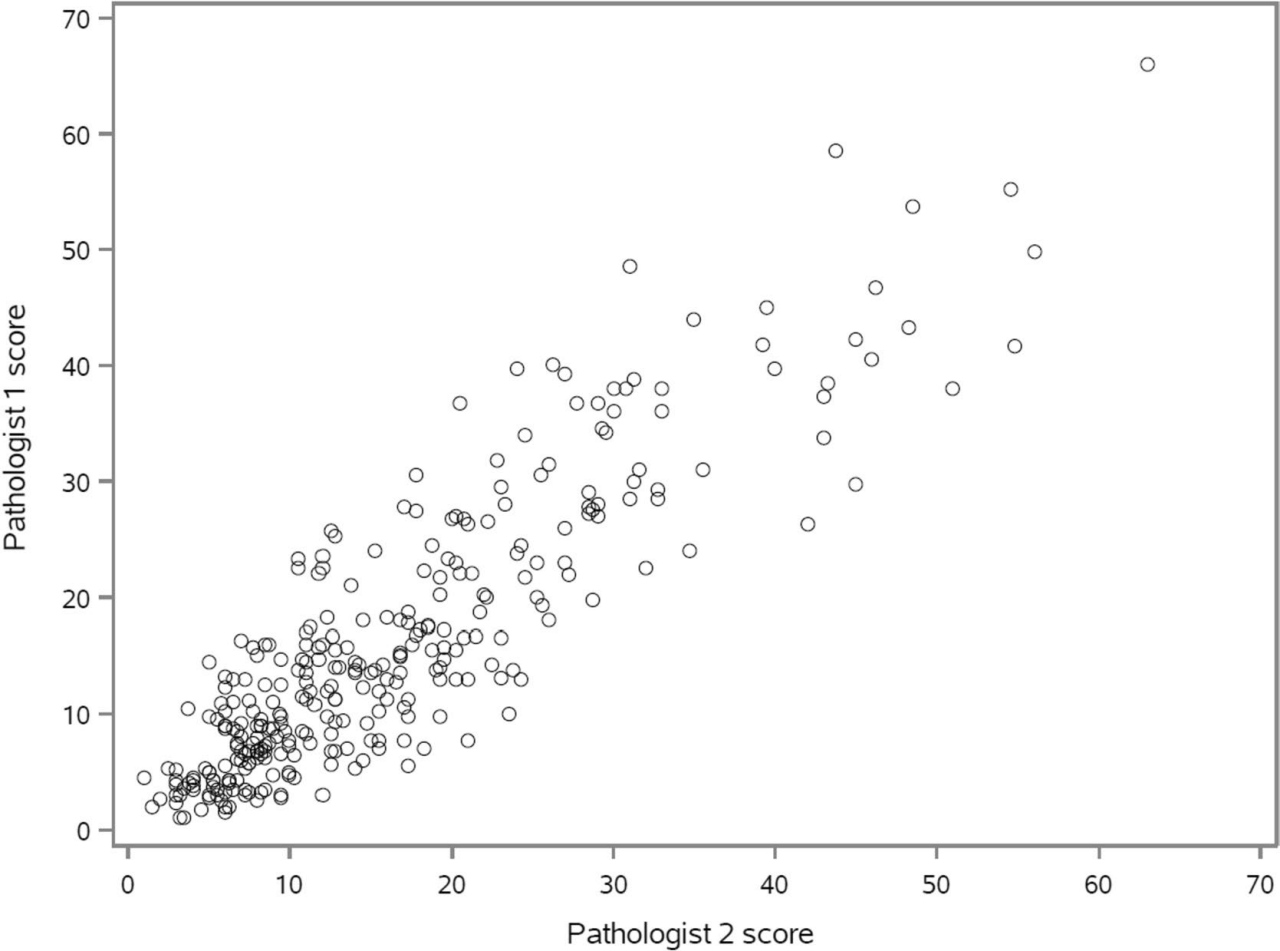
Scatter plot comparing pathologist 1 weighted Ki67 scores to pathologist 2 weighted Ki67 scores

A comparison of weighted Ki67 scores, using the global counting method, for the two pathologists is presented in Fig. 1. The ICC for Ki67 scores (log-transformed) by the two pathologists was 0.82 (95% CI 0.78–0.85); using cut points of ≤ 5, 6–29, 30+, the Kappa was 0.67 (95% CI 0.56–0.78). Using the IKWG guidelines, the scoring by pathologists took an average of 9–13 min per case. The ICC for IA vs pathologist 1 was 0.79 (95% CI 0.74–0.83); it was 0.76 (95% CI 0.71–0.80) for IA vs pathologist 2.

A comparison of reader scores (pathologist 1, pathologist 2, and image analysis) for Ki67 and 21 gene recurrence scores are presented in Table 2. Depending on the reader, 8.8–16.0% of our cohort had Ki67 ≤ 5% and 11.4–22.5% had scores ≥ 30%. Among patients with Ki67 scores ≤ 5% by pathologist 1 (*n* = 49, 16.0%), pathologist 2 (*n* = 27, 8.8%), or IA (*n* = 33, 10.7%), the percentages with RS < 26 were 91.8%, 92.6%, and 90.9%, for pathologist 1, pathologist 2, and IA, respectively. Among patients with Ki67 scores ≥ 30 by pathologist 1 (*n* = 41, 13.4%), pathologist 2 (*n* = 35, 11.4%), or IA (*n* = 69, 22.5%), the percent who had a RS ≥ 26 was 41.5% for pathologist 1, 51.4% for pathologist 2, and 27.5% for IA.

**Table 2:** Ki67 scores by reader and 21-gene recurrence scores (RS)

Reader	Ki67 scores	Total *N* = 307 (column %)	RS < 26	RS ≥ 26
			*N* = 267 (row %)	*N* = 40 (row %)
Pathologist 1	Weighted ≤ 5%*	49 (16.0)	45 (91.8)	4 (8.2)
	Weighted 6–29%*	217 (70.7)	198 (91.2)	19 (8.8)
	Weighted ≥ 30%*	41 (13.4)	24 (58.5)	17 (41.5)
	Weighted < 10%**	120 (39.1)	115 (95.8)	5 (4.2)
	Weighted < 20%***	217 (70.7)	202 (93.1)	15 (6.9)
Pathologist 2	Weighted ≤ 5%*	27 (8.8)	25 (92.6)	2 ( 7.4)
	Weighted 6–29%*	245 (79.8)	225 (91.8)	20 (8.2)
	Weighted ≥ 30%*	35 (11.4)	17 ( 48.6)	18 (51.4)
	Weighted < 10%**	111 (36.2)	105 (94.6)	6 (5.4)
	Weighted < 20%***	218 (71.0)	202 (92.7)	16 (7.3)
Image analysis	≤ 5*	33 (10.7)	30 (90.9)	3 (9.1)
	6–29*	205 (66.8)	187 (91.2)	18 (8.8)
	≥ 30*	69 (22.5)	50 (72.5)	19 (27.5)
	< 10**	81 (26.4)	77 (95.1)	4 (4.9)
	< 20***	176 (57.3)	162 (92.1)	14 (8.0)

*Cut points for percent staining recommended by IKWG [5]

**Cut points for percent staining used in POETIC

***Cut points for percent staining used in MonarchE

### Secondary analyses

Since other studies have used different Ki67 cut points, we also present results for cut points at 10% and 20%. Among patients with Ki67 scores of < 10% by pathologist 1 (*n* = 120), pathologist 2 (*n* = 111), or IA (*n* = 81), the percent with a RS of < 26 was 95.8% for pathologist 1, 94.6% for pathologist 2, and 95.1% for IA. Among patients with Ki67 scores of < 20% by pathologist 1 (*n* = 217), pathologist 2 (*n* = 218), or IA (*n* = 176), the percent with a RS of < 26 was 93.1% for pathologist 1, 92.7% for pathologist 2, and 92.1% for IA.

When analyses were restricted to patient subgroups based on tumor characteristics, we found that the percent of patients with Ki67 < 10% who also had RS < 26 was slightly higher among patients with PR > 10%; it was 98.2% for pathologist 1, 97.0% for pathologist 2, and 96.0% for IA. Among patients with low grade tumors < 2 cm the percent of patients with Ki67 < 10% who also had RS < 26 was 96.9% for pathologist 1, 94.9% for pathologist 2, and 97.7% for IA. When we used tumor grade to identify patients with low or high RS, we found that 97.2% of low-grade tumors had RS < 26. This increased to 99.1% when we restricted patients to PR > 10% staining.

## Discussion

In our study of early-stage breast cancer patients with favorable prognosis—women aged 50 years or older, with node-negative disease and ER positive, PR positive, HER2− tumors—we found that visual assessment of Ki67 IHC after undergoing IKWG training was moderately to strongly reproducible across two IHC pathologists (ICC = 0.82), with 8.8–16.0% having Ki67 scores ≤ 5% and 11.4–13.4% having scores ≥ 30%. In addition, we found that > 90% of patients with Ki67 scores ≤ 5% also had RS < 26. However, a large percent (50–70%) of those with Ki67 scores ≥ 30% by visual scoring also had RS < 26. Ki67 scoring concordance with RS was fairly similar across pathologists and with scoring by IA.

When following IKWG visual scoring guidelines, the recommended protocol was time consuming and challenging for our IHC pathologists who practice in a large integrated health care system with a centralized breast IHC service and high patient volume. Using the global counting method, the inter-rater reproducibility (ICC = 0.82) of our pathologists was slightly lower than that reported by the IKWG (ICC = 0.87) [5]. It is possible that with more practice, visual Ki67 reading might become more reproducible and faster, but the necessary time for each slide is likely too long in a high-volume setting or even for lower volume settings. Automated scoring using digital image analysis may be able to address this issue. The IKWG found high reproducibility of IA scoring for Ki67 IHC across laboratories using the same scanner (ICC = 0.89), although reproducibility was lower across sites using 10 different software platforms using 7 different scanners (ICC = 0.83) [5, 17]. When comparing scores from IA with visual scores, they observed slightly better concordance using the global (average of fields) vs. hot spot (maximum field) scoring method. In our study, scoring by IA used only the hot spot method while visual scoring by our immunopathologists only used the global method, as recommended by the IKWG. Thus, we were unable to examine whether the concordance for our immunopathologists vs. IA varied by global vs. hot spot method.

In their updated recommendations, the IKWG has concluded that visual scoring of Ki67 IHC could be used for ER+HER− stage I/II patients using ≤ 5% and ≥ 30% as clinical cut points such that results below and above these thresholds could be used to withhold or proceed with chemotherapy, respectively, without the need for more expensive multi-gene assays, such as the 21-gene RS [5]. However, they indicate this requires using a highly analytically validated assay and scoring system. Recent ASCO guidelines also suggest that these Ki67 cut points may be used in this clinical setting when patients do not have access to multi-gene assays [12]. Several clinical trials have used other cut points, but for different indicated uses. For example, POETIC used Ki67 < 10% in a study of neoadjuvant endocrine therapy [2], and MonarchE used Ki67 < 20% in a study of CDK4/5 inhibitors [18]. However, concerns regarding lack of standardized scoring and inter-rater and inter-laboratory reproducibility apply in these settings, as well [3].

There are examples of testing pathways for multi-gene assays in early-stage breast cancer. In the UK, the National Institute for Health and Care Excellence (NICE) recommends a two-stage strategy for testing ER+HER2−, node-negative breast cancer patients with a multi-gene assay [14]. First, a validated tool such as PREDICT or the Nottingham Prognostic Index is used to classify patients into low, intermediate, or high risk. Those with low-risk disease are unlikely to benefit from adjuvant chemotherapy. For those classified as intermediate risk, the multigene assays RS, EndoPredict, or Prosigna are recommended as options for guiding adjuvant chemotherapy decisions.

Our goal was to develop a simple testing pathway for RS using only Ki67 results but among a slightly more targeted population of early breast cancers with good prognosis (i.e., PR+ and age 50+ years as well as ER+HER−). While in our study over 90% of patients with Ki67 ≤ 5% had low RS, this was a very small subset (< 20%) of all the patients with low RS. Further, a substantial proportion of patients with Ki67 ≥ 30% also had low RS. Thus, using the recommended IKWG cut points for Ki67, even in a very targeted population, appears unlikely to be a very clinically useful testing pathway for multigene assays. Interestingly, in our study, concordance with low RS was slightly higher when we used the cutoff point of Ki67 < 10% or Ki67 < 20% and both of these cut points identified a substantially larger subset of patients with low RS than the ≤ 5% cut point. Thus, finding the most appropriate Ki67 cut points for a clinical decision may need further study and may continue to differ for different patient populations or treatment decisions.

We found that further restricting the study population to patients with PR > 10% staining marginally improved the correlation between Ki67 ≤ 5% and low RS. These findings are consistent with other studies that have used PR and Ki67 together to identify a subset of patients with good prognosis [19, 20]. When examining concordance of other tumor factors with RS, low tumor grade was the most concordant with low RS. This is also consistent with other studies (e.g., Paik [15]).

While we are unaware of other studies that have compared Ki67 IHC results following the IKWG guidelines (i.e., slides from core biopsy, training of pathologists for use of standardized scoring methods) with RS results from testing conducted by Genomic Health (recently ExactSciences), a number of other studies have compared Ki67 IHC results with RS results [6–11, 15, 21]. As in our study, others have observed discordance between Ki67 IHC and RS results based on various cut points. Some also have found that concordance improves when restricting patients to those with higher PR scores (e.g., Gluz [21]) or low-grade tumors (e.g., Paik [15]). In addition to discordance between Ki67 IHC and the RS, substantial discordance has been reported for comparisons across multigene testing [15], which has been accepted by the clinical community. Until there are studies of Ki67 IHC using IKWG guidelines that examine prognosis or response to chemotherapy as endpoints, concordance with RS or other multi-gene assays may be reasonable surrogates. However, it is unclear what amount of discordance between Ki67 IHC and these multigene assays would be acceptable to the clinical community.

Although we followed IKWG recommendations to use biopsy specimens for Ki67 IHC, which is also routine clinical practice for other IHC markers, this may have in part contributed to some of our observed discordance with RS, since RS was done on surgical specimens, as is typical in clinical practice. Studies have shown that intratumoral heterogeneity is common in breast cancer [22–24]. A recent study found Ki67 heterogeneity in 18% of sampled breast cancers [25]. While we considered RS to be the gold standard in our study, our findings suggest that Ki67 concordance with RS may vary by clinical factors such as tumor grade. Interestingly, it appears that combining clinical factors (age, tumor size and grade) with RS provides better prognostic information for treatment decisions than RS alone [26]. This would likely be true for Ki67 IHC, as well.

Our study has several strengths and weaknesses. To our knowledge, our study is one of the first to compare Ki67 scoring following IKWG guidelines with results from the 21-gene Recurrence Score assay, a currently NCCN recommended diagnostic for selecting patients for chemotherapy. While our health care and high-volume pathology system may not be generalizable to all settings, our pathology departments follow ASCO/CAP guidelines for processing specimens and our clinicians follow NCCN and other professional guidelines for patient management. We used the biopsy specimen for Ki67, as is recommended by IKWG, and had pathologists undergo IKWG web-based training and follow its scoring guidelines. We also examined multiple Ki67 cut points for their concordance with low RS (< 26) and we explored whether concordance might be improved among a more restricted patient population. Our eligible study population was restricted to patients who had testing by the 21-gene assay and did not include all women with ER+PR+HER2−, node-negative disease diagnosed at 50+ years. If those tested differed from those untested by factors we did not access (i.e., factors other than age, ER, PR, HER2, nodal status, grade) and these factors are also related to the concordance of Ki67 scores and RS, our findings may be biased. Another limitation is that we only examined reproducibility across two pathologists and results might differ if other pathologists had been included. However, these pathologists do the majority of our IHC scoring in our health care system. We also did not examine reproducibility of Ki67 scores across multiple IA platforms or laboratories, but the laboratory we used (NeoGenomics) is CLIA certified.

In summary, we found IKWG’s recommendation on Ki67 IHC visual scoring challenging in our real-world, high-volume setting, even with very experienced IHC pathologists and where IHC reading is centralized. Visual scoring results were fairly similar to results by IA and future studies will be needed to determine the extent to which inter-rater and inter-laboratory reproducibility can be maintained or improved by IA, which would also reduce pathologist time. However, the IKWG cut point of Ki67 ≤ 5% was only able to identify a small proportion of patients who could avoid RS testing based on recent IKWG recommendations. Future studies will be needed to determine whether using a higher Ki67 cut point, as well as including additional tumor features, such as PR > 10% or low tumor grade, could increase concordance and also identify a greater proportion of patients who could avoid RS testing. In the absence of prospective trials of Ki67 in ER+HER− stage I/II patients, studies will also be needed to determine the amount of discordance with RS or other multigene assay surrogates that would be acceptable to clinicians when trying to develop a testing pathway.

## Data Availability

The datasets generated during the current study are not publically available but are available from the corresponding author on reasonable request.
